# Common huntingtin-related genetic variation is associated with neurobiological and aging traits in humans

**DOI:** 10.1038/s41420-022-01114-1

**Published:** 2022-07-09

**Authors:** Alana N. Slike, Galen E. B. Wright

**Affiliations:** 1 Department of Pharmacology and Therapeutics, Rady Faculty of Health Sciences, University of Manitoba, Winnipeg, MB Canada; 2 Neuroscience Research Program, Kleysen Institute for Advanced Medicine, Health Sciences Centre and Rady Faculty of Health Sciences, University of Manitoba, Winnipeg, MB Canada

**Keywords:** Genetics, Neurological disorders

The conserved huntingtin gene (*HTT*) is known for its role in the neurodegenerative disorder Huntington disease (HD) [1]. This disease is caused by expansions of the polyglutamine (polyQ) tract in exon one of *HTT*, primarily encoded by CAG repeats [1, 2]. Repeat length predicts the age of HD onset, with longer lengths associated with earlier HD onset on average [3]. CAG repeat length is variable in humans, individuals affected by HD have an expansion of 36 or more CAG repeats [4]. Therefore, we read with interest the recent study by Iennaco et al., which examined the function and evolutionary aspects of non-pathogenic *HTT* CAG repeats [5].

Notably, Iennaco et al. found that selection in humans favoured longer CAG tracts, suggesting that an increase in the *HTT* polyQ tract length, below the pathogenic threshold, may provide evolutionary advantages. Specifically, it was proposed that longer non-pathogenic CAG tracts increase neurogenic potential, alter transcription networks responsible for neuronal function and contribute to evolutionary fitness. These findings support the notion that non-pathogenic HTT plays a vital neurological role in humans [5].

Currently, knowledge regarding the specific role of non-pathogenic HTT protein is limited. Nonetheless, previous studies have implicated HTT in several biological processes, including autophagy, vesicular transport and development [1]. *HTT* exhibits a high level of genetic constraint for loss of function mutations, providing additional evidence biological importance in humans [6]. Furthermore, rare deleterious mutations in *HTT* cause Lopes-Maciel-Rodan syndrome, a neurodevelopmental disorder with a clinical presentation similar to Rett syndrome [6].

We, therefore, aimed to assess the contribution of common *HTT* genetic variation to diverse traits in humans to gain further insight into the role of *HTT* in both human health and disease. To accomplish this, we assessed fine-mapped signals from large-scale genome-wide association studies (GWAS) where *HTT* has been mapped with high confidence as being the most likely causal gene. The unbiased nature of these studies can help identify previously unappreciated relationships and functions of the gene, thereby informing the biological underpinning of the *HTT* selective pressure observed by Iennaco et al.

GWAS data was extracted on 8 March 2022 from the Open Targets Genetics database v7 (22.02). This database is a comprehensive repository of genetic associations from the UK Biobank and GWAS literature, containing important metrics to prioritize candidate causal variants and genes at trait-associated loci. Notably, machine learning-based models, trained on comprehensive genetic and functional genomic features, perform fine-mapping of significant association signals via the locus-to-gene (L2G) model, with scores ranging from 0-1 (higher scores represent stronger evidence for a gene being causal) [7].

The database currently contains information for 50,543 studies, including summary statistic information for 8317 human GWAS, representing 132,893 independent genome-wide significant loci. We filtered these data to detect signals where *HTT* is predicted to be the most likely causal gene at this locus (i.e., an L2G score of 0.5 or greater for *HTT*). We estimated the number of independent signals (i.e., haplotypes) by pruning index variants using r^2^ = 0.5 in the 1000 Genomes European super-population with LDLink SNPclip [8].

We identified 28 unique trait associations with 23 unique genetic variants at the *HTT* locus. After removing redundant associations, such as blood cell type measurements, ten traits and six unique variants remained (Table 1). These traits include cognitive and non-cognitive processes, as well as longevity-related traits. The machine learning model, L2G, predicted *HTT* to be the most likely causal gene for these trait associations (mean L2G_*HTT*_ = 0.63). Our analyses identified trait associations for common genetic variation attributed to *HTT* that were captured via three independent signals (i.e., haplotypes)﻿.

**Table 1:** Prioritized *HTT* human trait GWAS associations confirm the critical role of the gene in both health and disease.

Reported trait (PMID)	Study N	Index	Signal (tag variant)^a^	Annotation^b^	Effect AF EUR^c^	GWAS index *P* value	GWAS index effect (95% CI)	L2G_*HTT*_
LDL cholesterol levels x short total sleep time interaction (31719535)	61,548	rs2298969	Haplotype one (rs61348208)	Intronic	G: 0.49	4.0 × 10^–9^	NA	0.76
Educational attainment: years of education (30595370)	455,000	rs363096	Haplotype two (rs363096)	Splice region	C: 0.59	1.0 10^–10^	NA	0.76
Noncognitive aspects of educational attainment (33414549)	510,795	rs363096	Haplotype two (rs363096)	Splice region	C: 0.59	1.0 × 10^−12^	0.05 (0.04–0.06)	0.70
Highest math class taken (30038396)	811,539	rs113928896	Haplotype three (rs113928896)	Intronic	T: 0.14	2.0 × 10^−9^	0.02 (0.01–0.02)	0.63
Parental lifespan (30642433)	500,193	rs61348208	Haplotype one (rs61348208)	Intronic	T: 0.39	6.0 × 10^−9^	0.23 (0.15–0.31)	0.61
Aging traits: health span, parental lifespan or longevity (32678081)	837,415	rs61348208	Haplotype one (rs61348208)	Intronic	T: 0.39	3.0 × 10^−8^	NA	0.60
Depression (30718901)	807,553	rs7685686	Haplotype one (rs61348208)	Intronic	G: 0.43	6.0 × 10^−15^	0.98 (0.98–0.99)^d^	0.59
Frailty index (34431594)	175,226	rs82334	Haplotype one (rs61348208)	Intronic	C: 0.32	3.1 × 10^−10^	−0.02 (−0.03 to −0.02)	0.58
Gastroesophageal reflux disease (34187846)	602,604	rs7685686	Haplotype one (rs61348208)	Intronic	G: 0.43	1.1 × 10^−8^	0.95 (0.96–0.98)^d^	0.55
Frequency of tiredness / lethargy in last 2 weeks (NA)	350,580	rs61348208	Haplotype one (rs61348208)	Intronic	T: 0.39	6.6 × 10^−9^	−0.01 (−0.02 to −0.01)	0.55

In total, 28 unique traits had an L2G_*HTT*_ score greater than 0.5, with ten select associations (six unique variants) attributed to the *HTT* gene presented in the table. These ten select associations represent three independent signals (i.e., haplotypes).

Table ordered by L2G_*HTT*_ score.

*AF* allele frequency, *EUR* 1000 Genomes European super-populations, *L2G* Open Targets locus-to-gene model score, *NA* not applicable.

^a^LDlink SNPclip pruned signals using an r^2^ of 0.5 in the 1000 Genomes Project European superpopulations.

^b^Ensembl VEP ‘most severe’ annotations.

^c^1000 Genomes Project European superpopulation effect allele frequency.

^d^Odds ratio (all other effects are beta estimates).

Haplotype one, captured by tag variant rs61348208, was responsible for the majority (i.e., 70%) of the prioritized *HTT* associations. This signal includes four intronic *HTT* index variants, with the effect alleles associated with increased *HTT* gene expression in skeletal muscle in GTEx. This haplotype was associated with multiple traits related to longevity, including frailty index and parental lifespan. This includes the results from a large-scale lifespan GWAS (*N* = 500,193) performed by Timmers et al. [9]. Of interest, rs61348208 (associated with increased *HTT* expression), was found to be a lifespan-extending allele, increasing lifespan between 0.23 and 1.07 years [9]. Similarly, another study by Timmers et al. examined aging traits via a multivariate meta-analysis of GWAS identified traits and found that this *HTT* signal was significantly associated with years of good health and lifespan [10]. Furthermore, this haplotype captured a signal from a GWAS meta-analysis for the number of adverse health events which occurred during an individual’s life (i.e., frailty index) [11]. Specifically, the *HTT* increased expression allele corresponded with the minor effect GWAS allele and was associated with a lower frailty index (Beta = −0.02) [11]. *HTT* trait associations identified here suggest a role in longevity and a beneficial effect of the *HTT* gene product, strengthening the case for positive selection for the gene in human populations.

Haplotype two was captured by a splice region variant, rs363096, and was associated with educational attainment (EA) traits. Genetic aspects of EA have been shown to correlate with cognition, wellness, health outcomes, and longevity [12]. A large UK Biobank GWAS (*N* = 455,000) found the rs363096 index variant was associated with EA (L2G_*HTT*_ = 0.76) [13]. While this initially points to a role in cognition, further analysis of the data by Demange et al. suggest non-cognitive aspects of EA may drive this signal [14]. This study examined GWAS of EA and cognitive test performance to determine non-cognitive traits of EA via subtraction. In this regard, the previous index rs363096 variant was associated with non-cognitive aspects of EA. This trait showed correlations with neurobiological phenotypes, including personality and psychiatric traits, and associations displayed enrichment in neuronal cell types [14].

Additionally, the non-cognitive dataset was positively correlated with longevity and explained most genetic correlations between EA and lifespan [14]. This is consistent with the trait associations found in haplotype one, supporting *HTT* influencing longevity. However, the exact traits driving this *HTT* association with this complex phenotype need to be resolved, with further studies exploring the potential impact of alternative splicing in neural cells. Together, these trait associations suggest that *HTT* plays a role in non-cognitive neurological function, lending support for a positive neurogenic role of *HTT*.

Lastly, haplotype three is also independently associated with an EA-related trait. This signal originated from a comprehensive meta-analysis-based GWAS (*N* = 811,539) of EA, representing 71 cohorts [15]. In this study, the *HTT* intronic index variant (rs113928896) was associated with the highest level of math an individual has taken. However, while *HTT* was the most likely causal gene (L2G_*HTT*_ = 0.63), the G protein-coupled receptor kinase, *GRK4*, also had a high L2G score (L2G_*GRK4*_ = 0.54). Therefore, further functional genomic studies are needed to confirm if *HTT* is driving this signal.

Notably, our study is the first systematic, unbiased assessment of common *HTT*-related genetic variation in human health and disease. As a result, we have gained insight into the non-pathogenic function of *HTT* by identifying potential roles of *HTT* outside of HD by analyzing information for the gene at the population level in humans. While we were unable to directly assess associations between *HTT* CAG repeat lengths and human traits due to GWAS technology limitations, the generation of whole-genome sequencing information in these cohorts will allow for this to be assessed in the future. Further, future studies must be performed to validate these results with functional genomics to further elucidate the non-pathogenic role of *HTT*.

## Data Availability

The datasets analyzed during the current study are available in the Open Targets repository, https://www.opentargets.org/.
